# The Differential Patho-Anatomical Diagnosis in Glanders*The following article was drafted especially for the instruction of the Chief Veterinary Police officers in Germany, and is now published for the first time.

**Published:** 1886-04

**Authors:** 

**Affiliations:** The Imperial Veterinary Institute, Berlin, Germany


### ART. IX.—THE DIFFERENTIAL PATHO-ANATOMICAL DIAGNOSIS IN GLANDERS.*

BY PROFESSOR SCHUTZ,

*Of the Imperial Veterinary Institute, Berlin, Germany.*

The fact of the great frequency with which we find primary pulmonary glanders mentioned in the animal statistics with regard to animal pest in cases of horses that have been premptorily slaughtered by the Veterinary Police on account of real or suspected glanders, has long been a matter of general attention.

In the Fifth Annual Report of the Technical Veterinary Commission of Prussia, one may see that it is the view of the majority of the authorities, that primary pulmonary glanders occurs in one-half or even two-thirds of the cases of glanders without there being any indication of complications either in the nasal cavities or skin.

These assertions do not, however, agree with actual experience, and hence lead us to conjecture that other complications than those of glanders have frequently led to a mistaken diagnosis, and that in consequence the number of cases of unquestionable glanders must be less than those given in the statistics. Consideration must also be given to the fact that many official veterinarians, scarcely ever mention any other form than pulmonary glanders in their reports, but, at the same time, have no other grounds for their conclusions than the presence of a few isolated caseated or calcified noduli in the lungs.

* The following article was drafted especially for the instruction of the Chief Veterinary Police officers in Germany, and is now published for the first time.

In order to bring the question of the prevalence of primary pulmonary glanders to some definite conclusion, it was ordered by the minister of agriculture that the lungs of all horses that had been killed by the authorities in which those making the autopsies considered the above complication to exist, should be sent to the Pathological Laboratory of the Veterinary School for a detailed examination. In accordance with this order there were sent to the school (from the 24th of February to the 10th of October, 1882,) the lungs of 127 horses; of these 92 were decided to have the pathological lesions of glanders, and 35 to be free from the same. The following remarks are based largely upon the results of the study of these organs.

Glanders is a disease that finds its origin in a contagious principle, which transplants itself solely in the equine, and is transmitted to other species of animals, including man, by means of accidental infection only. The specific pathological product in glanders is a small nodulus being about the size of a millet seed, which is composed largely of round cells and a more or less dense stroma or connective tissue. These noduli must be, therefore, classed among the connective or granulation tissue neoplasms. This tissue is soft and contains more or less fluid in its meshes, and is of a yellowish-white color. The more rich in cellular elements, such neoplasms are, the more clouded or pearly in color do they appear. The fresh nodulus in glanders is gray in color and consists of small round cells; the older ones are of a yellowish color and contain, at the same time, larger cellular elements. These nodular neoplasms undergo necrobiotic degeneration which frequently leads to the formation of cavities where they are situated in the midst of paranchymatous organs; when seated superficially, as in the nasal mucosæ or the skin, such degeneration leads to the formation of ulcers. When fatty metamorphosis occurs in these noduli, they are clouded yellow in color; this cloudiness has frequently led to the noduli being looked upon as (genuine) tubercles.

Attention must be called to the fact that many pulmonary diseases in the horse are characterized by the presence of circumscribed productions of the most variable character and form; it is also too frequently the case that many veterinarians designate every variety of nodulus met with in the lungs as tubercle. Hence it is that, even in the present day, we often find the noduli in glanders mistaken for genuine tubercles. The name tubercle is not only a descriptive term, *i. e.*, with reference to form alone, but has also a distinct scientific importance ; it is used to designate very small noduli (tubercula) of a certain kind. Even though the products in tuberculosis bear a certain resemblance to those in glanders, still from the etiological point of view they vary essentially from one another. It must also be borne in mind that genuine tuberculosis is an exceedingly rare disease in the horse, and that the fresh tubercle is pearly and transparent, the older, opaque and whitish, while the nodulus in glanders is, when fresh, grey; but later on, yellow; and finally bears a strong resemblance to small abscesses. The nodulus in glanders resembles the tubercle, however, in that they frequently form nests or conglomerate masses, and break down, especially when superficially situated, as in a mucosa.

It is incorrect, however, to compare the ulceration of a nodulus in glanders with the bursting of an abscess. Whenever an abscess comes to development, the tissues are destroyed, in the place of which is formed a cavity filled with pus, which finally bursts, leaving a distinct interruption in the continuity of the tissues involved ; the nodulus in glanders undergoes, on the contrary, a gradual process of ulcerative destruction, the material which escapes from the same being the detritus or remnants of destroyed tissues. As this softening or ulcerative degeneration does not at once complicate the entire nodulus, we find the base and circumference of the neoplasm the seat of neoplastic granulations presenting a lardaceous appearance. Such an ulcerated nodulus gradually cleans itself from its necrobiotic elements, leaving nothing but a most superficial ulceration which is frequently difficult of recognition and having the peculiar form which has given to it the name of "lenticulor ulcer."

These lenticulor ulcers have too frequently been described as erosions, an entirely erroneous expression, for the latter is nothing more or less than the circumscribed removal of the epithelial covering from underlying tissues. In lenticulor ulcerations, however, not only the epithelium, but also a portion of the tissues under it, undergo necrobiotic destruction

The development of the nodulus in glanders and the progressive process of ulcerative destruction which it undergoes can be best studied in the nasal mucosa. In a short time, there develops a new nodulus and ulcer, either in the circumferences or in the deeper seated tissues of the primary one at some distance from it; from the coalescing of such neoplasm and sequential extension of the ulcerative processes, ulcerated surfaces developed of variable extent and form, which are known as secondary ulcers. The latter are characterized by their swollen irregular edges and uneven base, and by the presence of newly developed and small noduli that have not yet undergone destruction. This nest-like development indicates that the primary nodulus, or ulceration, exerts an irritative action upon the adjoining tissues, which unites in them the development of similar disturbances, and indicates that the contagious elements of glanders proliferate in the primary noduli and penetrate into the adjoining tissues by means of the capillary circulation. It is not necessary that the secondary processes develop in the immediate vicinity of the primary; they can appear at a distance from the source. This manner of extension of the pathological processes in glanders is especially frequent in the respiratory tract, when they can extend to the inferior part of the nasal cavities or the sinuses of the head, the œsophagus, larynx, trachea, bronchial tubes, or even the lungs. The ulcerations may be either superficial or profound, according to their situation in the tissues, but scarcely any tissue is free from them. They often extend to the perichondrium in the nasal septum or the valvular covering of the eustachian tubes, or the cartilages of the larynx, trachæ and bronchial tubes, and to the periosteum and underlying osseous tissues in appropriate localities, causing necrobiosis in all such parts. It is not an unfrequent occurrence that perforation of the septum nasi results therefrom, or that cicatricial contractions and deformations occur in other parts of the respiratory tract. The processes of glanders in the mucosæ are often connected with simple inflammatory disturbances in their vicinity. The noduli and ulcers are frequently surrounded with a nucleus of injected blood vessels which renders their recognition essentially easier than it otherwise would be. In most cases, we find the adjoining mucosa in a more or less catarrhal condition, swollen and secreting an aqueous, purulent or hæmorrhagic mass. The seat and extent of the catarrhal irritation decides the quantity and nature of the secretion in horses diseased with glanders more than the ulcerative disturbances in the anterior portion of the respiratory tract. The bronchial mucosa is frequently the seat of such excessive irritation that their luminea is completely filled with a muco-purulent mass. The catarrhal processes often extend from the nasal cavities to the frontal and lateral sinuses of the head, causing them to become filled with a more or less dense secretion of a similar character. They can also extend from the pharynx through the eustachian tubes to the guttural pouches. When the irritation thus caused is very violent the secretions acquire an hæmorrhagic character. These simple inflammations, *i. e.*, such that are only secondarily due to the glanders irritant, often complicate the more profoundly situated tissues. The mucosa, especially, the submucosa of the nasal cavities, becomes intensely swollen and odematous. In the larynx, such processes frequently cause "œdema glottidis."

When the infiltrative processes have reached a certain depth in the tissues, we often find a purulent perichondeitis in the septum nasi, less often in the larynx, but especially frequent on the processes vocales. These disturbances frequently lead to necosis and perforation in the complicated parts, or to serious cicatricial retractions.

The ulceration in glands, either primary or secondary, often heal through cicatization, so that we can see hundreds of such scars in the trachea. These cicatrices have more or less stellate form, but possess no constancy in this direction however. When the ulcerative processes extend equally in all directions from a primary nodulus, the healing first begins at the centre, while the neoplastic and ulcerative disturbances extend at the peripheries. These large ulcerations can also heal; which they do by the ordinary wound-healing granulation processes; they are frequently the seat of more or less extensive hæmorrhages. This cicatrization does not, however, always occur in a harmless manner; for, as already said, all sorts of malformations upon the parts complicated may occur, even to the utter destruction of the entrance to or lumen of the diseased cavities or tubes. It is unfortunate that definite healing seldom results for the development of new noduli, and ulcers most frequently come to pass either underneath the newly developed cicatricial tissue or in its circumferences; similar processes also develop within the elements of the cicatrices themselves, giving the impression that the latter were also the object of deuteropathic invasion, hence cicatrization is not always the termination in glanders, but simply a part of the same.

The frequent recidives that occur in glanders are the cause of the chronic and indurative character frequently seen in simple inflammatory processes which are represented by superical and more profoundly seated disturbances; the mucosæ of the respiratory tract are most frequently the seat of such wart or plaque-like excrescences.

It has been already mentioned that the specific processes in glanders can extend from the nasal cavity to the adjoining sinuses; the most frequent occurrence is, however, a chronic catarrh in the latter. The mucosa, with which they are lined, secretes such immense quantities of a muco-purulent material that the cavity is either filled thereby, or can, even in very chronic cases, become gradually somewhat distended. The perivateum of living cavities often participates in the disturbances which are characterized by the development of verrucose or more less extended neoplastic productions.

The processes in the skin as well as the mucosæ are invariably accompanied by complications of the lymphatic system. The latter one of a secondary nature, and bear direct connection with those parts from which the glands receive their lymph. The contagious principle is taken up by the lymphatics in and around the primary diseased localities and by them, conveyed to the first lymph glands in their course, and from these the irritans is still further conveyed over the system. When the disturbances in the septum nasi are limited to one side, we find that the submaxillary, pharyngeal and other lymphatics upon that side only become diseased; the same is true with regard to glander complications beginning on one or the other extremities. The diseased glands become hypertrophied, and upon section present a more or less refracting surface of a reddish or greyish color. Later on, they become dryer and the cut surface appears less smooth; in the latter we observe small white spots or striæ, which correspond to the swollen follicles or the thickened inter-follicular tissue. When the irritation is very severe (acute glanders) we often find the glands the seat of more or less extensive hæmorrhages.

This is the first or simple hyperplastic stage. Following on this the glands become more dense and the cut surface is of a greyish-white, reddish-grey, or red-white color. The gland appears to be made up of cellular (medullary) tissue, its cut surface being somewhat like velvet, on account of the projection of the swollen follicles.

This stage is known as that of "medullary hyperplasia." In neither of these stages, which of themselves have nothing characteristic of glanders, does the cellular hyperplasia attain the degree of development which frequently occurs under other irritative conditions in the nasal mucosa, which are bound with complications of the neighboring lymph glands. These glands in glanders seldom acquire a size beyond that of a walnut. It is also to be remarked that the individual nodes of the glands become somewhat flattened by mutual pressure, but do not become adherent to one another; at this period, the capsule of the gland is generally unchanged also and the skin covering the gland still unattacked. In the living animal, one can frequently notice that the glands are somewhat painful on pressure.

If the glanders processes in the skin and nose heal, the supply of irritating material is thus shut off from the lymphatics and the glands become smaller again, though not assuming their original size. In general, however, this does not occur, for, as already said, in most cases this disease is characterized by the gradual extension of its disturbance though healing in the primary affected parts is frequent. It is also well known that the enlarged condition of the glands can go on increasing through the development of neoplastic elements in the interstitial tissue, or stroma of the same by which they become intensely hard and undurated; these processes extend to the capsule and periadenoid tissue frequently causing their attachment to the overlying skin, or adjoining parts.

Among the specific occurrences in the glands must be mentioned the development of noduli; the earliest changes which are capable of macroscopical demonstration are small circumscribed points of a greyish or greyish yellow color; they may be either disseminated over the substance of the gland or accumulated in nests, and be found either in the medullary or cortical substance of the gland. No matter how much of the gland may be assumed by these specific disturbances, we shall always find the other parts in a condition of cellular hyperplasia of a simple irritative character. The yellow spots become larger and larger, and either undergo purulent degeneration, or become clouded, desiccated (Necrobiosis) and form caseous conglomerates or noduli. Such abscesses, or conglomerates may be frequently found in the midst of a hypertrophied gland. The specific irritant having gained access to the gland, exerts continuous action which leads to the development of chronic, indurative conditions.

As in all other specific diseases, so in glanders, we must divide the pathological disturbances into the primary, or specific, and secondary, or simple inflammatory processes. They are active processes, the one class may be compared to those which may follow any form of irritation; the other, on the contrary, possess peculiarities which are specific to glanders. When lymph glands are in a hyperplastic or indurated condition, we must not assume that they, of themselves, offer anything peculiar to our consideration; the cause, is the specific agent, which interests us; for by the latter alone can we decide whether the case before us is glanders or not. The specific affection speaks to us by its own peculiarities.

In glanders it is seldom that we find the entire gland complicated by processes peculiar to that disease; as a rule single parts or lobuli of the glands are more affected than others. Suppurative processes in or around the lymph-glands that finally escape outwardly are very rare in glanders. It must be remembered that simple suppurative inflammatory processes can, on healing, leave the glands in a condition strongly resembling those we have become acquainted with in glanders. The contents of such abscesses can desiccate and become cheesy, or peri-œdnoid indurations may occur by which the glands become hard, and more less attached to surroundings.

When, however, we observe, in specific disease of the lymph-glands in glanders that other centres of disease occur, and that large sections of the glands become diseased, and that a viscid purulent mass forms the contents of the swollen lymph-glands and that the cells early undergo disintegration, forming thereby a fatty detritus, the cavities being filled with a mush-like mass of fat-granules; that the noduli do not undergo complete caseation, but remain a sort of soft yellowish centre; we have before us certain valuable points for the differential diagnosis. If there still remains some doubts about the correctness of our conclusions, they may all be removed by giving special attention to the conditions at the primary seats of disease. We frequently find a mild form of fatty-metamorphosis in lymph-glands that are in a condition of simple hypertrophy. A cross section of the same appears, broken by whitish yellow points or striæ, while the remaining parts of the gland are in a grey hyperplastic condition. In this form of retrograde metamorphosis, the hyperplastic part of the lymph-glands is composed of fatty detritus also. If the glands have already suffered certain changes, being grey or blackish in color and indurated as well, which is often the case in the bronchial glands, the cross section then presents a peculiar variegated appearance.

We often find in lymph-glands, more especially in those in the vicinity of the bronchials, than in the submaxillaries, calcarious noduli of variable dimensions and shape, that are generally surrounded by a firm capsule. These masses can easily be removed from the latter, leaving their cavity, or surroundings, distinctly visible. A cross section of these noduli present a lamellated structure. Although it is not always possible to give a decision as to the primary nature of these noduli, especially as to whether they are of entozoöic origin, still many veterinary observers have ascribed to them an etiological connection with glanders without sufficient reason. It is not explainable why the noduli of glanders should become calcified in this isolated manner. It is highly probable, on the other hand, that noduli that are not so easily removed, and that have not a striated structure, are connected with glanders. But a calcification of the noduli in glanders has not been, by any means, proven in these organs, the objects must, at present, be looked upon as of an etiologically doubtful origin. Large calcified masses, occupying distinct sections of a gland, must be considered as a metamorphosis, occurring in retained purulent material. We have seen that the glanders noduli undergo dissolution in course of time, and are transformed into a mass of detritus, capable of being absorbed; this does not disturb the contagious principle in this disease, however. This mass is taken up by the circulation as well as the lymphatics ; in the former case they cause direct polution (infection) of the blood —glanders dyscrasis—, the latter has been partially proven experimentally by the injection of the blood from horses with glanders into healthy ones ; the same fact is also proven by the appearance of metastatic processes in distant parts of the organism of the same animal. The lungs are, in general, the seat of such metastatic, or secondary processes in glanders. It is, therefore, evident that the necroscopist must distinguish sharply between primary and secondary processes in this disease. The skin and superior parts of the respiratory tracts are most frequently the seats of these protophatic processes. In the latter, it is generally the lower section of the nasal cavity, the edges of the sublimated bones, the nasal canals, and their posterior openings, and the larynx, where the glanders processes first develop. It has been assumed that the contagious principle in glanders gains access to the lungs by means of the inspired air ; *i.e.*, that the lungs can be the seat of primary processes ; an hypothesis which finds its support in many reports from official veterinarians. It is incorrect to assume that this is a general or frequent occurrence, for in all the cases upon which this report is based, it was not possible to find one that gave support to the theory of primary pulmonary glanders. One either found distinct disturbances in other organs,such as the above-mentioned, that accompanied the lungs, or they were mentioned in the reports from the official veterinarians. Secondary pulmonary glanders is, however, a very common occurrence. It is self-evident that when a glanders-dyscrasis occurs, secondary pulmonary processes must develop in other organs, such as the kidneys, liver, heart, muscles, bones, etc. Such a dyscrasis is not a permanent condition by any means, as when the infectious elements gain access to the blood they must soon be again removed by natural processes, from the fact that they find a resting place in certain tissues or organs, where they develop metastatic centers, or pass away with the secretions —urine, etc. This latter was proven by numerous experiments. If the disease continues in certain parts it is self-evident that this tendency to metastatic processes must also continue. It is a very common occurrence that the processes in the anterior respiratory passages and skin heal, that the lymph glands undergo a retrograde metamorphosis and retract, and that the secondary metastatic disturbances only come to recognition after the lapse of no inconsiderable time. This period is often spoken of as "latent glanders," that is, the disease is present in invisible organs—pulmonary glanders. If horses should be killed at this time, we do not find any striking pathological changes, except those in the lungs; it is frequently the case that slight changes in the anterior respiratory organs heal without leaving easily recognizable cicatrices, and the tumefactions in the lymph glands also disappear quite as often, especially when the inflammatory processes are of a non-specific character. Such cases have led to the mistaken assumption of the existence of primary pulmonary glanders. It is also well known that this latent, or hidden, form of glanders often gives rise to secondary infection and the development of new local centers and of suspicious phenomena, which render the disease again open to ocular demonstration. It is possible that such cases have led to the assumption of the spontaneous origin of glanders.

The specific glanders noduli in the lungs always appear in numbers, never as single phenomena; they are somewhat more than miliary in size, and are at first gray and moist, but later become yellow and dry in character. They either lie sub-pleural, so that they can be seen through the same, or in the substance of the lungs; they develop in the stroma, or connective tissue of these organs which separates the lobuli one from another. The fresh noduli are surrounded by an irregular zone of inflamed paranchymatous tissue, the circumferences of which are not sharply defined. These zones are hyperæmic, moist, atalectatic, and present a smooth surface or section, though they are sometimes granulated—fibrinous pneumonia. We may frequently see the nodules differentiated quite sharply from these pneumonic surroundings by a red ring of tissue. We find here the usual combinations of specific (glanders) and simple inflammatory processes, and in passing the fingers over the pleura of such a lung we not only feel the noduli specific to glanders, but also the pneumonic centres surrounding them. The number of these noduli is very variable; in some lungs they are present in thousands, sometimes disseminated, sometimes in nests, or conglomerates, so thick together that the infiltrated pneumonic centers coalesce, so that large sections of the lungs are tranformed into a dense and atalectetic mass. The noduli and their surroundings soon undergo certain changes. The gray noduli become opaque and yellow, or yellowish-white, surrounded by a transparent gray, and vascular tissue, which can be traced to its connections with the interlobular tissue and the septa of the alveoli. These yellow centers are slightly caseous, but do not present a thoroughly desiccated appearance; their circumferences are not regular, but are interrupted by the extension of processes of a like character with the fibrous surroundings, so that we cannot remove these noduli, even with the aid of the point of a knife, without tearing the tissues in which they are situated. When confluence of numerous noduli has taken place, we find that large sections of the lungs have been transformed into a dense mass of gray or grayish-white indurated tissue which encloses the necrobiotic noduli in its midst; upon sections of the same the latter appear as small yellow centers surrounded by a sort of capsule, or as conglomerates of the same color, with irregularly-marked outlines. At other times these centers are of a purulent character, surrounded by infiltrated pulmonary tissue, which is often complicated by softening disturbances that began in the specific products of glanders, giving rise to the formation of cavities of variable size and form, that are filled with a purulent or mush-like mass. New eruptions may be seen in the vicinity of these centers which are of essential value in recognizing the specific nature of the disturbance. At other times we find the disintegrated mass surrounded by a pale gray layer of fibrous tissue which forms a sort of capsule; the cross section of such spots reveals the existence of cavities and canals, filled with purulent and necrotic material. The ulcerative disturbances frequently lead to the erosion of blood-vessels and sequential hæmorrhages; the latter are, however, often prevented by the formation of thrombi in the vessels—a very common occurrence in the lungs in glanders. Small embolic centers may be mistaken for freshly-developed glanders noduli; they vary in size from milliary to that of a pea, and are filled with a yellowish or clouded mass, surrounded by a hyperæmic umbus of a dark red color, and having a smooth surface on section. The hyperæmic tissue surrounding the noduli in glanders is never black-red, and not so wide in extent, and does not contain so much fluid blood as the hæmorrhagic. Acute broncho-pneumonia centers may be also mistaken for those peculiar to glanders; the same are small isolated complications, or they may be seen in groups, and are of a red or reddish-gray color, projecting somewhat above the cut surface of the lung; the bronchial tube presents itself as a small, yellowish point in the midst of pneumonic infiltration; if one pour water gently upon such points we can easily demonstrate the presence of the bronchial tubes which will appear a small round hole, out of which has been washed the mass with which it was filled. It has frequently happened that centers of ordinary pneumonia that have undergone a form of gangrenous dissolution have been mistaken for those of glanders. Pneumonia in the horse often occurs in a multiple form, *i.e.*, in numerous inflammatory centers distributed over the substance of the lungs; they are at first hyperæmic and glancing, but later on becomes dry (anæmic) and yellowish or yellowish-white in color. Necrobiosis of the pulmonary tissue frequently occurs which leads to the development of a sort of sequestor, or of a mass of broken-down tissue enclosed in a sort of capsule, which marks the line of demarcation from still healthy tissue. It is in this way that the so-called abscesses in the lungs are formed. Fresh ones are immediately surrounded by intact or infiltrated lung tissue, while older ones are enclosed by indurated tissue, making a sort of capsule. The bronchial lymph glands are at the same time in a condition of cellular or fibrous hyperplasia. The fresher these conditions, the more easily can they be distinguished from others, because we cannot find the gray or yellowish noduli of glanders in the diseased portion of the lungs at such times. The differential diagnosis is, however, possible later on, because it is very seldom in glanders that we find either single or several infiltrated lobuli separated, as it were, by these gangrenous processes in the direction of the interlobular striæ of connective tissue, while such is the rule in pneumonia. Further, the necrotic tissues in pneumonia, which were previously very full of blood, do not give up their color, as in glanders, but retain a very marked yellowish-red color. Finally, one must always have recourse to a critical study of the conditions in other organs.

The frequent presence of calcified noduli of a size corresponding to those of glanders, have often led to mistaken conclusions. They are generally round, with sharply-defined limits, and enclosed in a very delicate, transparent capsule of connective tissue, from which they can easily be removed. The inner surface of this capsule is perfectly smooth. It has never been possible to find any adhesions between these objects and the bronchial tubes; cross sections of these calcified noduli shows a striated structure, they are often distributed over the lungs in great numbers, as well as in the bronchial glands, but less frequently in the sub-maxillaries; they seem to have a special predilection for the liver. *These things have no connection with glanders.*

Bronchitic, peribronchitic, and bronchostatic noduli have been frequently mistaken for those of glanders of an old date. It is natural that this error should have been made when we remember the extension which the idea of primary pulmonary glanders has attained among veterinarians.

These bronchial noduli are the product of chronic inflammatory processes in the parieties of the bronchials; they appear in certain forms; some develop into the lumen of the tube causing obstruction—bronchitis proliferons—in other cases aside from the thickening of the walls of the tube, we find the secretion of a purulent fluid, causing distension of the tube—(bronchitis chronica catarrhalis). When the irritation processes extend to the tissues surrounding the tubes, we have a peribronchitis leading to the development of connective tissue and apparent thickening of the walls of the air tubes. The purulent mass in the tubes frequently accumulates and becomes caseous—bronchitis caseous—causing complete obstruction; at other times, this mass calcifies and we find the lime-like material enclosed by the bronchial wall. These irritative processes generally occur in and around bronchial tubes of the smallest calibre; they may be quite extensive, or more or less sharply circumscribed; in the latter case, the tubes look as if possessed by small noduli along their extent—bronchitis and peri-bronchitis nodosa. This circumscribed or nodular form has been the cause of many mistaken conceptions and assertions on the part of veterinary authors.

They present themselves as small round bodies, of the size and form of millet seed; they are often numerous, but at other times are but sparsely distributed over the lungs. In the case of bronchitis proliferons, these noduli have a gray color, the lumen of the tube can often be distinguished upon making a cross section of the nodules. In bronchitis catarrhalis and caseosa we find the center of the nodules filled with a purulent or caseous material surrounded by pearly-gray tissue, which corresponds to the cheded walls of the bronchial tube, so that we see that what was apparently a nodulus, is in reality nothing but the cross-section of an indurated bronchial tube. When the caseous material with which the bronchials are filled at such a point has become calcified, we find a small kernel of lime making up the center of such a nodulus; the same may be round or elongated in form, according to its extent, and is easily removed.

Bronchostasis is a gradual and progressive process by which the lumen of the tube becomes greater, and in the horses comes most frequently in the smaller tubes of the anterior and lower, or central lobes of the lungs; they generally occur in multiple form, seldom isolated. According to the thickness of the wall of the tube, we speak of them atrophy and hypertrophy, and according to form, as cylindrical, sacculated, serpentine, etc. In every case of bronchostasis we find bronchitis in one form or another. Outside of them pneumonia processes frequently come to development. The circumscribed, or sacculated form often appears as noduli, being about the size of a pea, but cross section soon reveals their true nature.

It should be absolutely impossible to mistake any of the conditions for the specific ones of glanders, though one or all of them may appear in the same lung at the same time with those of glanders, or at times when there is no evidence of the latter. In fact, they are not uncommon in old horses in all varieties and degrees of development.

In all glanders noduli the purulent or caseous mass in the center is always in immediate relation with pulmonary tissue; in these bronchial conditions never; the bronchial wall invariably being demonstrable as a demarcating tissue. In the latter, the contents can always be removed, leaving the wall intact, in glanders never.

Again, of differential diagnostic value is the fact that the noduli in glanders are seldom of the same age; besides old and anæmic, we find fresh ones that are easily recognizable. Again, as a rule, the noduli in glanders are of metastatic origin, and in doubtful cases, the exact examination of the other portions of the respiratory tract, as well as body, will certainly lead to a safe conclusion.

Finally, we have to mention those chronic inflammatory or indurative processes in the lungs which generally find their origin in a chronic pleuritis or bronchitis, or begin directly in the peri-alveolar tissue. Such processes frequently lead to the development of circumscribed centers of a more or less nodular character, but very variable in size, being sometimes as large as a hen's egg; they may be frequently found in close proximity to one another, or they may coalesce, and form large irregular, circumscribed and indurated masses in the lungs; they are especially frequent in the anterior and middle lobes; such indurated spots give manifest resistance to the knife on cross section, being very dense and hard, and present a white or gray color. If such changes are in the vicinity of the pleura, the latter is also thickened, white and non-transparent; adhesions between the pleuræ are then frequent. Sometimes these nodes have but little retractive tissue in them when they present a refulgent and transparent appearance on section. Obstruction of the lumen generally follows chronic processes upon, in, or around the bronchial tubes; the same leads to atalectatic conditions of the parts supplied with air by the obstructed bronchials. In anæmic animals, such sections of the lungs are quite pale in color. If the atalectatis is of considerable duration we find that atrophy of the pulmonary tissue results. These changes occur principally in the anterior lobes, and in the lower and middle portions of the lungs.

When these atalectatic or atrophied portions of lungs become the seat of chronic pneumonic processes we find that they remain small, soft and singularly anæmic. It is self-evident that both conditions can be present at the same time. When it was mentioned that, as a rule, bronchostasi occurred in the smaller bronchi, it was meant that they did not occur on the larger, for it is well known that the latter are subject to a great variety of malforming changes.

It is not an uncommon occurrence to find that ulcerative (destructive) processes have originated in the distended portion of the tube, and finally caused perforation of the wall and pneumonic disturbances of both an ulcerative and indurative character. As the progress of the same can be traced, there is no need of mistaking them for glanders disturbances as has been frequently done.

Of the thirty-five lungs in which glanders did not exist, six were complicated with chronic pneumonia, nine with simple bronchostasis and four with the latter and connective pneumonia. In the other ninety-two lungs, aside from the specific phenomena of glanders, chronic pneumonia existed in five cases, simple bronchostasis five times, and complicated with chronic pneumonia thirteen times.

It is therefore plain that we have to distinguish quite sharply between the specific productions in the lungs that are peculiar to glanders, and due to its specific cause, and those secondary complications which, while frequently due to the action of the same cause, may either antidate the development of the specific products, or even occur entirely without their ever happening.

It was a mistake to have classed indurative conditions in the lungs among the specific and direct products of glanders, and from this has proceeded the doctrine of primary pulmonary glanders. This name is entirely misleading for these conditions, as they are not primarily connected with the etiological moment in glanders.

It has been our endeavor to draw the attention of official veterinarians to the necessity of distinguishing sharply between what is primarily and solely due to glanders, and what follows is secondary disturbances; also to the necessity of paying the strictest attention to all parts of the organism when considering the lungs or any internal parts.

